# Climate change weakens the tie between weather and mast seeding

**DOI:** 10.1038/s42003-021-02033-0

**Published:** 2021-04-13

**Authors:** Caitlin Karniski

**Affiliations:** Communications Biology, https://www.nature.com/commsbio

## Abstract

Climate change has been shown to affect the interannual variation and synchrony among individuals in seed production of masting trees, yet the proximate mechanisms driving these patterns remain unclear. A recent study by Michał Bogdziewicz and colleagues shows that the relationship between weather cues and seed initiation weakens in European beech as warming increases, resulting in progressive asynchrony of seed maturation. This study emphasizes the vulnerability of the relationship between environmental cues and forest reproduction to climate change.

Climate change is known to impact tree reproduction, and specifically seed production, worldwide. These effects may be especially pronounced for masting species, which reproduce through synchronized reproductive events at variable intervals and are tied to annual variation in weather. Masting plays an important role in ecosystems and for forest regeneration, so understanding environmental determinants of masting dynamics is critical. Climate change has been shown to affect masting by impacting interannual variation and synchrony in seed production among individuals, but the mechanisms that drive these patterns have been unclear.

**Figure:**
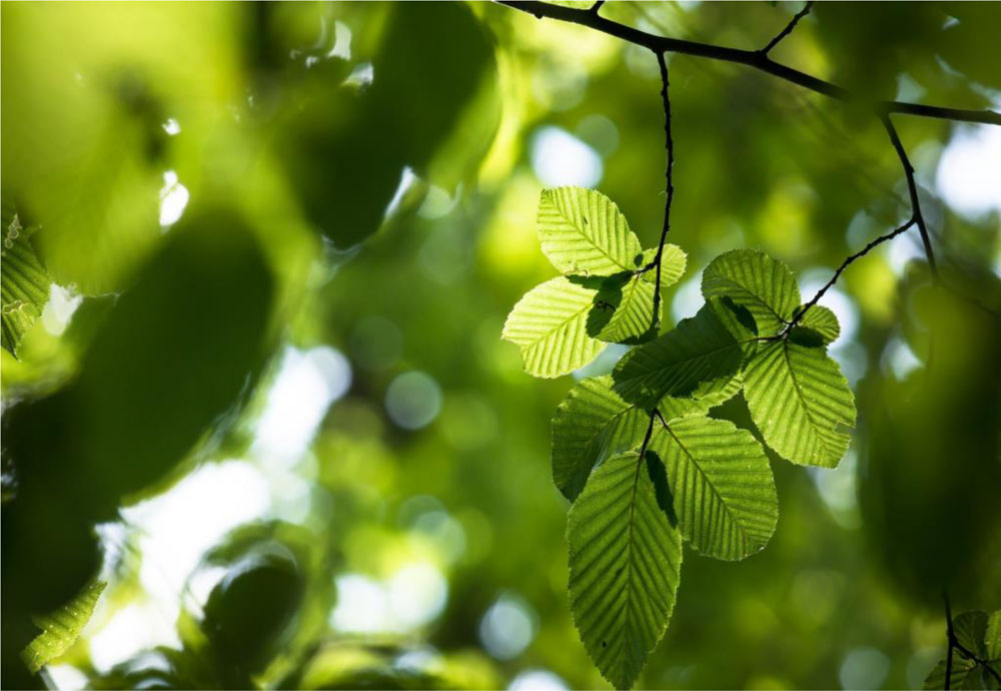
Thomas B/Pixabay

A recent paper^1^ led by Michał Bogdziewicz of Adam Mickiewicz University uses a 39-year dataset of European beech *Fagus sylvatica* L. in England to investigate how climate change impacts the transitions among seed development phases over time. Despite an increase of ~1 °C in maximum temperature over the course of the study period from 1980 to 2018, the authors found that the seasonal peaks in the relationship between weather and seed production occurred at relatively the same time each year. However, they also found that the frequency of temperature anomalies, as defined by a temperature more than one standard deviation from the long-term mean, increased. Positive summer temperature anomalies increased over time, while the frequency of negative summer temperature anomalies decreased. In early years of the study, summer weather anomalies led to mast flowering. Specifically, seed initiation, the overall number of seeds produced, was highest when a relatively cold summer was followed by a relatively warm one. However, this relationship weakened over time and by the end of the study period in 2018, this pattern of a cold summer preceding a warm summer was no longer a significant predictor of seed initiation. Accordingly, the synchrony of seed initiation across sites as affected by summer temperatures also diminished over time. Throughout the earlier years of the study, the synchrony of seed initiation was comparable to that of seed maturation, though in the later years, the synchrony of seed maturation had become reduced relative to that of seed initiation.

The authors characterize these weakening relationships between weather and seed development and its timing as a breakdown in the weather cueing process, leading to asynchronous flowering and further resulting in offset synchrony of seed maturation. Though the authors conclude that density-dependent pollen limitation was not responsible for masting synchrony, pollen coupling still exacerbates the patterns seen here with desynchronized flowering. Taken together, these results demonstrate how the seed development cycle is affected by climate change and further reveals the vulnerability of the relationship between environmental cues and forest reproduction when threatened by climate change.

## References

[1] Bogdziewicz M (2021). Climate warming causes mast seeding to break down by reducing sensitivity to weather cues. Glob. Chang. Biol..

